# Clinical Epidemiology of Pediatric COVID-19 Delta Variant Cases From North Sumatra, Indonesia

**DOI:** 10.3389/fped.2022.810404

**Published:** 2022-04-01

**Authors:** R. Lia Kusumawati, Inke Nadia Diniyanti Lubis, Meutia Ayuputeri Kumaheri, Ariel Pradipta, Kiatichai Faksri, Mutiara Mutiara, Anuraj H. Shankar, Tryna Tania

**Affiliations:** ^1^Department of Microbiology, Faculty of Medicine, Universitas Sumatera Utara, Medan, Indonesia; ^2^Microbiology and Biomolecular Laboratory, Murni Teguh Memorial Hospital, Medan, Indonesia; ^3^Department of Paediatrics, Faculty of Medicine, Universitas Sumatera Utara, Medan, Indonesia; ^4^Genomik Solidaritas Indonesia Laboratory, Jakarta, Indonesia; ^5^Department of Microbiology, Khon Kaen University, Khon Kaen, Thailand; ^6^Research and Diagnostic Center for Emerging Infectious Diseases, Khon Kaen, Thailand; ^7^Centre for Tropical Medicine and Global Health, Nuffield Department of Medicine, University of Oxford, Oxford, United Kingdom; ^8^Eijkman-Oxford Clinical Research Unit, Jakarta, Indonesia

**Keywords:** SARS-CoV-2, COVID-19, pandemic, children, infection

## Abstract

The Delta variant of SARS-CoV-2 (severe acute respiratory syndrome coronavirus-2) dominated the coronavirus disease 2019 (COVID-19) pandemic in 2021. Here we report the Delta variant among pediatric cases in North Sumatra, Indonesia, from June to July 2021. Whole-genome sequencing (WGS) from 18 new COVID-19 pediatric patients showed that six were B.1.459 and six were B.1.466.2, known variants in Indonesia in clade 20A. Six were the Delta variant B.1.617.2 of clade 21A, with five on one branch and one on a distant branch consistent with that patient's geographic separation, suggesting at least two introductions to the region. Variants tended to be spatially clustered, and four children with Delta variant had an adult infected household member, all of whom had lower real-time polymerase chain reaction cycle threshold (Ct) values compared with the child. No temporal trends were observed for Ct. These data support a paradigm shift with children being highly susceptible to the Delta variant and a priority for vaccination.

## Introduction

The Delta variant (B.1.617.2) of severe acute respiratory syndrome coronavirus-2 (SARS-CoV-2), the cause of coronavirus disease 2019 (COVID-19), was first detected in India in December 2020 and dominated the pandemic in 2021 due, in part, to its higher transmissibility. (1). The Ministry of Health of the Republic of Indonesia reported the first Delta variant case on April 3, 2021 in Jakarta, and it quickly spread to other provinces (2), leading to the highest numbers of new cases (*n* = 350,273) and deaths (*n* = 7,118) in any country as reported by the World Health Organization (WHO) on July 20, 2021 (3). By July 23, 2021, Indonesia reported an escalating trend of new COVID-19 cases for 9 consecutive weeks. Of 34 provinces, 32 experienced an increase in new cases compared with the previous week, with six provinces reporting more than a 150% increase, including North Sumatra (238%) (4, 5). With 25 provinces reporting presence of the Delta variant, the surge pushed the pandemic impact in Indonesia to 4,100,138 infections with 133,676 deaths by September 1, 2021; the spike in Delta cases had mostly declined (6).

In North Sumatra, the Delta variant was first reported in July 2021 from imported cases among 18 crew members of a ship docked in North Sumatra province (2). However, a rapid increase of cases in North Sumatra began in the last week of June, suggesting introduction from another source, too. By August 5, 2021, North Sumatra ranked as the seventh highest province with 21,876 cases and 1,581 deaths (7). Despite this, there were no reports of the Delta variant in children in North Sumatra. Globally, the variant had caused an increase in total COVID-19 cases, hospitalizations, and deaths in children (8), contributing to 1.7% to 2% of confirmed cases (9). Unfortunately, reports indicate that the proportion of affected children is much higher in Indonesia, comprising 12.5% of cases with a 3–5% case fatality rate. In addition, SARS-CoV-2 transmission in children continues to present an increasing trend (10).

To the best of our knowledge, no study has reported on the presence, or the clinical relevance, of the Delta variant in children from a low- and middle-income country (LMIC). Therefore, we report herein findings from an investigation of the emergence of the Delta variant in pediatric COVID-19 patients from North Sumatra, Indonesia.

## Methods

### Settings

**Figure 1:**
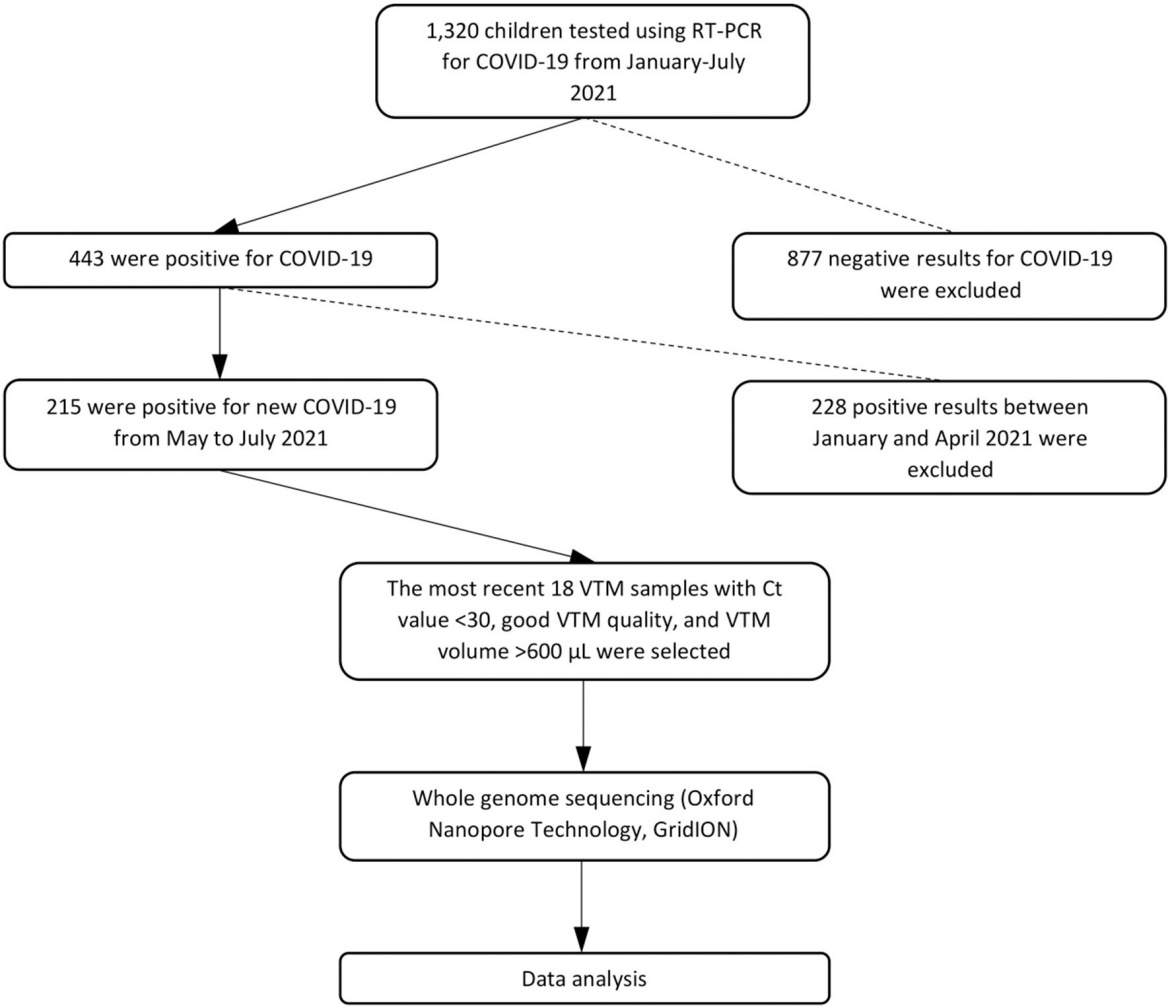
Study flowchart.

From May 14, 2021, to July 11, 2021, we collected nasopharyngeal–oropharyngeal swabs into viral transport media (VTM) from 18 suspected COVID-19 pediatric outpatients at Murni Teguh Memorial Hospital (MTMH) in Medan (Figure 1). Swabs were analyzed at the MTMH Microbiology Laboratory for SARS-CoV-2 by real-time reverse transcription–polymerase chain reaction (RT-PCR). Positive specimens were selected for whole-genome sequencing (WGS) after excluding those with cycle threshold (Ct) value >30 or with degraded VTM quality indicated by yellowish color or VTM volume <600 μL. Ct value indicates the number of RT-PCR cycles until the fluorescent intensity exceeds background levels for a positive reaction. The Ct value has a linear relationship with the logarithm of the initial copy number, with a lower Ct value suggesting greater amounts of virus in a swab (11). Demographic and laboratory data were retrieved from hospital electronic medical records. Data from pediatric patients at MTMH from January to July 2021 were also collected and analyzed, and Ct values and positivity rates were evaluated to determine trends in pediatric cases. The study flowchart can be seen in Figure 1. The study protocol was approved by the Ethics Committee of the Faculty of Medicine, University of Indonesia—Cipto Mangunkusumo Hospital (protocol no. 21-05-0535).

### SARS-CoV-2 Ribonucleic Acid Isolation

Ribonucleic acid (RNA) was extracted using an automated magnetic bead–based process (Tianlong Nucleic Acid Extractor, Xi'An Tianlong Science and Technology Co., Ltd., ShaanXi, Xi'an, Republic of China) and kit (Tianlong Viral DNA & RNA Extraction Kit T014H, Xi'An Tianlong Science and Technology Co., Ltd., ShaanXi, Xi'an, Republic of China) for SARS-CoV-2 according to the manufacturer's instructions. The isolation process was done with 200 μL VTM added to lysis buffer, followed by nucleic acid binding, washing buffer, and finally elution of RNA for RT-PCR.

### RT-PCR

RT-PCR was done using either the Tianlong Novel Coronavirus (2019-nCoV) Nucleic Acid Detection Kit or the JN Medsys ProTect™ COVID-19 RT-qPCR Kit 2.0 or the Detection Kit for 2019 novel (2019-ncov) RNA (PCR-fluorescence probing) DaAn Gene Co., Ltd. of SunYat-sen University, and following the manufacturer's instructions. In brief, the process was done by adding the master mix solution and 5 μL of sample to wells of a 96-well plate for RT-PCR. The RT-PCR steps were RT at 45°C for 15 min, RT inactivation and DNA polymerase activation at 95°C for 2 min, denaturation at 95°C for 3 s, and annealing and extension at 55°C for 30 s. The Tianlong kit targeted two genes, *N* and *RdRP*, to confirm SARS-CoV-2; the JN Medsys kit targeted only the *N* gene; and the DaAn Gene kit targeted *N* and *ORF1ab*. Tianlong, JN Medsys, and DaAn Gene included a sample extraction internal control of RNaseP or Cy5. A Ct value <40 for each gene was defined as the cutoff to confirm a positive result for SARS-CoV-2.

### Whole-Genome Sequencing

The 18 RT-PCR–positive VTM samples were sent to Genomik Solidaritas Indonesia Laboratory, Jakarta, Indonesia, and underwent quality control for eligibility before WGS. Samples were extracted using MGIEasy Nucleic Acid Extraction Kit (Shenzhen, PRC) and MGISP960 automated system per the manufacturer's instruction. RNA was converted into cDNA using LunaScript RT Supermix (LunaScript™) using the reverse transcriptase enzyme. RNA purity was checked from the 260/280 ratio (normal range for RNA 1.9–2.0) and 260/230 ratio (normal range 2.0–2.2). Genome enrichment was done using the protocol developed by the ARTIC Network for Oxford Nanopore Technologies (ONT). SARS-CoV-2 cDNA sample concentration was evaluated with a Qubit Fluorometer 4 (Thermo Fisher) using the manufacturer's standard protocol for HS DNA. The next step was SARS-CoV-2 genome enrichment with the ARTIC PCR Tiling protocol using V3 primers. The ONT GridION device was used to conduct WGS. One negative control using nuclease free water instead of cDNA, as well as one positive control, was included in the sequencing run. The positive control used was synthetic RNA Control 2 (GenBank Reference MN908947.3) supplied by Twist Biosciences (12). WGS data of these 18 SARS-CoV-2 samples have been deposited with GISAID ID EPI_ISL_3208058, EPI_ISL_3208059, EPI_ISL_3208060, EPI_ISL_3208061, EPI_ISL_3208062, EPI_ISL_3208063, EPI_ISL_3208064, EPI_ISL_3208065, EPI_ISL_3208066, EPI_ISL_3208067, EPI_ISL_3208068, EPI_ISL_3208069, EPI_ISL_3208070, EPI_ISL_3208071, EPI_ISL_3208072, EPI_ISL_3208073, EPI_ISL_3208074, EPI_ISL_3208086.

### Bioinformatics and Data Analysis

Base calling was performed using Guppy on the MINKnow software v.5.1.1 with the high accuracy model (ONT, UK) on an Ubuntu v18.04 virtual machine running an emulated Nvidia T4 GPU. Sequencing data were then demultiplexed using Guppy Barcoder (MINKnow v5.1.1) with a custom arrangement of the barcodes and with the option “barcodes_both_ends” and a minimum barcoding score of 50 at both ends to produce FASTQ files. The reads were mapped to the reference genome for Wuhan-Hu-1 (GenBank accession reference MN908947.3) using minimap2 (v.2.18-r1015). The mapped bases in BAM format were trimmed at the primer regions according to ARTIC software v. 1.3.0. The trimmed reads were then used for variant calling with Medaka software v.1.4.3. FASTA files were analyzed by Pangolin version 3.1.5 and Nextclade 0.13.0 prior to submission to GISAID. The SARS-CoV-2 phylogenetic tree was constructed with Nextclade v.1.5.4, and descriptive statistics were used to describe the results (13). Additional analyses of associations between variant types and Ct values, temporal patterns, or other characteristics were carried out with SAS 9.4 (Cary, NC, USA).

## Results

During this study period, new COVID-19 pediatric cases at MTMH comprised 7.3% of all new COVID-19 cases (childre*n* = 215 vs. adults = 2,953) from May 1, 2021, to July 31, 2021. Of 215 new pediatric COVID-19 cases, the most recent 18 VTM prior to July 12, 2021, the initiation date of the WGS study, stored at our laboratory with Ct value <30, volume >600 μL, and no change in VTM color were sent for WGS. In this study, we only performed WGS focused on pediatric COVID-19 cases, and we did not perform WGS on adult cases.

For the 18 pediatric patients with SARS-CoV-2 WGS, 12 were female (66.7%), and 10 were between 5 and 18 years old (55.6%). Fourteen were from Medan, the capital city of North Sumatra Province, with two from outside Medan but within the Province and two from outside the province. Three variants were detected, with six (33.3%) being the B.1.617.2 Delta variant of concern, six being B.1.466.2, and six being B.1.459 (Table 1). Clinical information of the 18 children with WGS was not available, but they were presumed to be mild cases as they were not hospitalized.

**TABLE 1:** Demographic data of 18 pediatric patients.

**Category**	**Total**	**%**
**Gender**		
Female	12	66.7
Male	6	33.3
**Age**		
<1 year	1	0.06
1– <5 years	7	38.9
5– <18 years	10	55.6
**City**		
Medan	14	77.8
Outside Medan	4	28.6
**Variant**		
Delta	6	33.3
B.1.466.2	6	33.3
B.1.459	6	33.3

WGS results (Table 2) revealed six patients with the Delta variant who were 1, 5, 6, 8, 10, and 14 years of age, with four females, five from Medan, and one from outside North Sumatra province. Four of six patients with B.1.466.2 were 1 year of age, and the rest between 3 and 14 years, with three females and five from Medan. Patients with B.1.459 were 4, 9, 10, 12, and 15 years of age, with one being 8 months of age, five males, and four from Medan.

**TABLE 2:** Whole-genome sequencing results from 18 children with SARS-CoV-2 infection in North Sumatra, Indonesia.

**Patient's ID**	**Age**	**Gender**	**Patient's origin of area**	**Specific name (PANGO)**	**Variant name (WHO)**	**First detected in**	**Date detected**
1	12 years	Female	Medan Johor, North Sumatra	B.1.459	Others	··	14 May 2021
2	15 years	Female	Karawang, West Java	B.1.459	Others	··	17 May 2021
3	1 year	Male	Sidikalang, North Sumatra	B.1.466.2	Others	··	19 May 2021
4	9 years	Female	Medan Perjuangan, North Sumatra	B.1.459	Others	··	2 June 2021
5	14 years	Female	Medan Timur, North Sumatra	B.1.466.2	Others	··	2 June 2021
6	4 years	Female	Medan Johor, North Sumatra	B.1.459	Others	··	3 June 2021
7	1 year	Male	Medan Johor, North Sumatra	B.1.466.2	Others	··	4 June 2021
8	1 year	Female	Medan Johor, North Sumatra	B.1.466.2	Others	··	4 June 2021
9	1 year	Female	Medan Johor, North Sumatra	B.1.466.2	Others	··	4 June 2021
10	10 years	Female	Pancur Batu, North Sumatra	B.1.459	Others	··	17 June 2021
11	14 years	Female	Medan Selayang, North Sumatra	B.1.617.2	Delta	India	25 June 2021
12	6 years	Female	Medan Petisah, North Sumatra	B.1.617.2	Delta	India	25 June 2021
13	1 year	Female	Medan Petisah, North Sumatra	B.1.617.2	Delta	India	25 June 2021
14	8 months	Male	Medan Polonia, North Sumatra	B.1.459	Others	··	25 June 2021
15	8 years	Female	Medan Barat, North Sumatra	B.1.617.2	Delta	India	4 July 2021
16	3 years	Male	Medan Timur, North Sumatra	B.1.466.2	Others	··	4 July 2021
17	5 years	Male	Medan Denai, North Sumatra	B.1.617.2	Delta	India	5 July 2021
18	10 years	Male	Pineleng, North Sulawesi	B.1.617.2	Delta	India	11 July 2021

**Figure 2:**
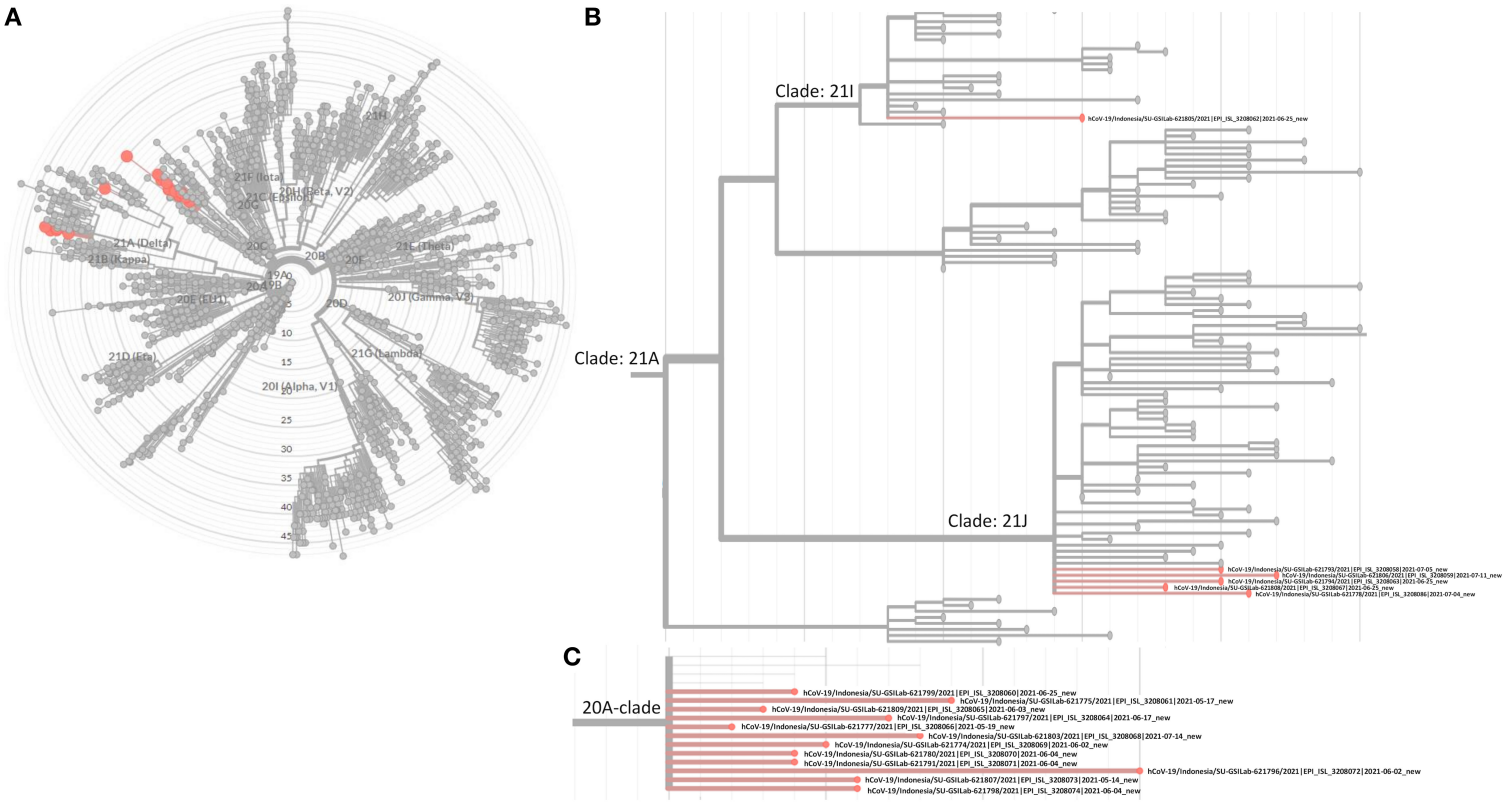
Phylogenetic tree built with Nextclade v1.5.4. **(A)** Highlighted 18 viruses as reported in Table 2. **(B)** Focused view of where the 6 Delta variants are located under the 21A clade (Delta variants). **(C)** Focused view of the other 12 viruses under clade 20A.

The SARS-CoV-2 phylogenetic tree (Figure 2) showed the detected variants could be divided into two clades, 20A and 21A (Figures 2A,B). Clade 20A included B.1.459 and B.1.466.2, previously known common variants in Indonesia, and all fell within the same branch. For clade 21A, the majority of the Delta variants (5/6) were on the same branch, suggesting they were closely related and of similar origin. However, one was on a more distant branch suggesting a different origin from the others, an observation consistent with its geographic location in a southwestern ward of Medan that was separate from other Delta variant cases (Figure 4). This would be consistent with two independent and early introductions of the Delta variant to the Medan region.

**Figure 3:**
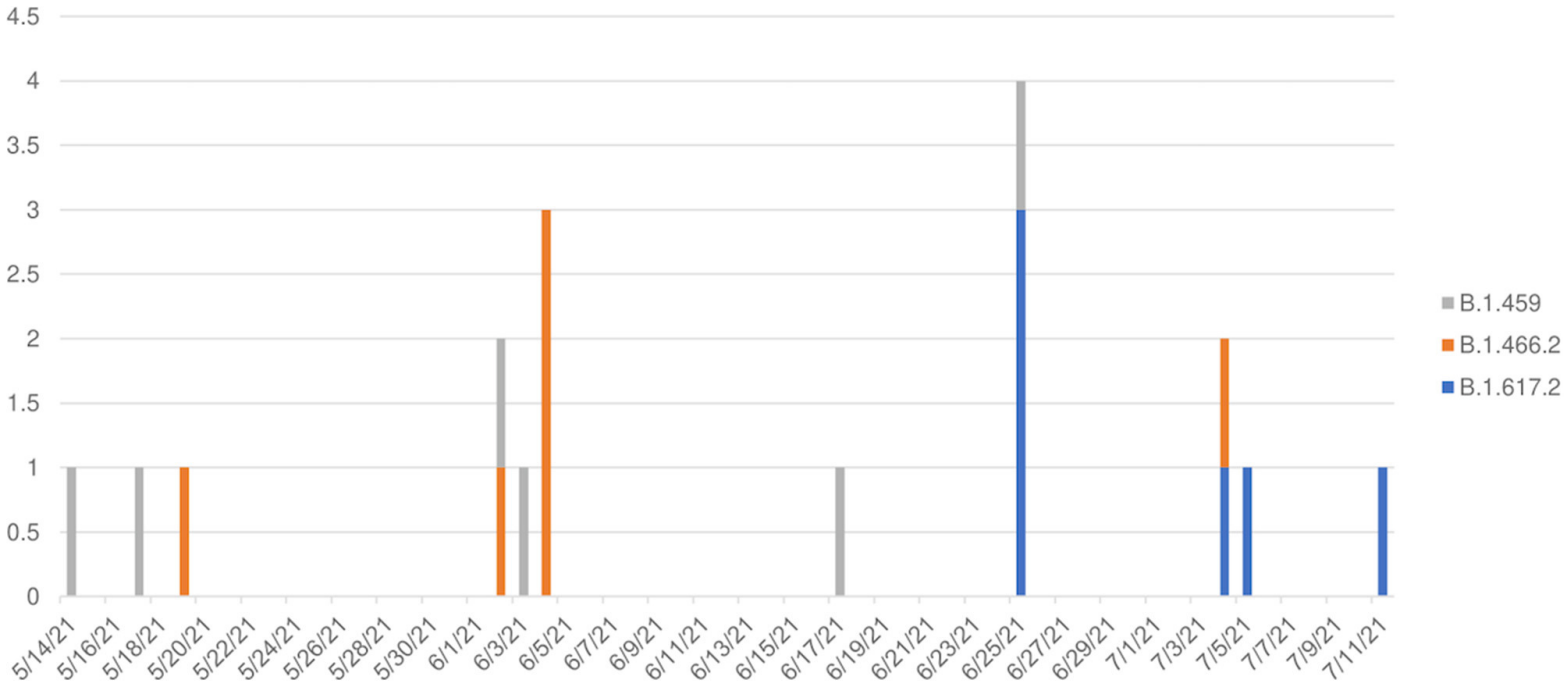
Temporal pattern of variants among 18 children with COVID-19 in North Sumatra, Indonesia. (*X* axis: date of detection (d/m/y); *Y* axis: number of cases).

The transition of predominant variants from B.1.459 and B.1.466.2 to Delta B.1.617.2 was observed from May 14 to July 11, 2021 (Figure 3), wherein Delta comprised 0/10 variants prior to June 25, but was 6/8 variants thereafter, a significant temporal shift (Kruskal–Wallis H test, *p* = 0.0115), suggesting 75% (95% confidence interval [CI] = 34%−97%) of pediatric cases in this time period were Delta.

**Figure 4:**
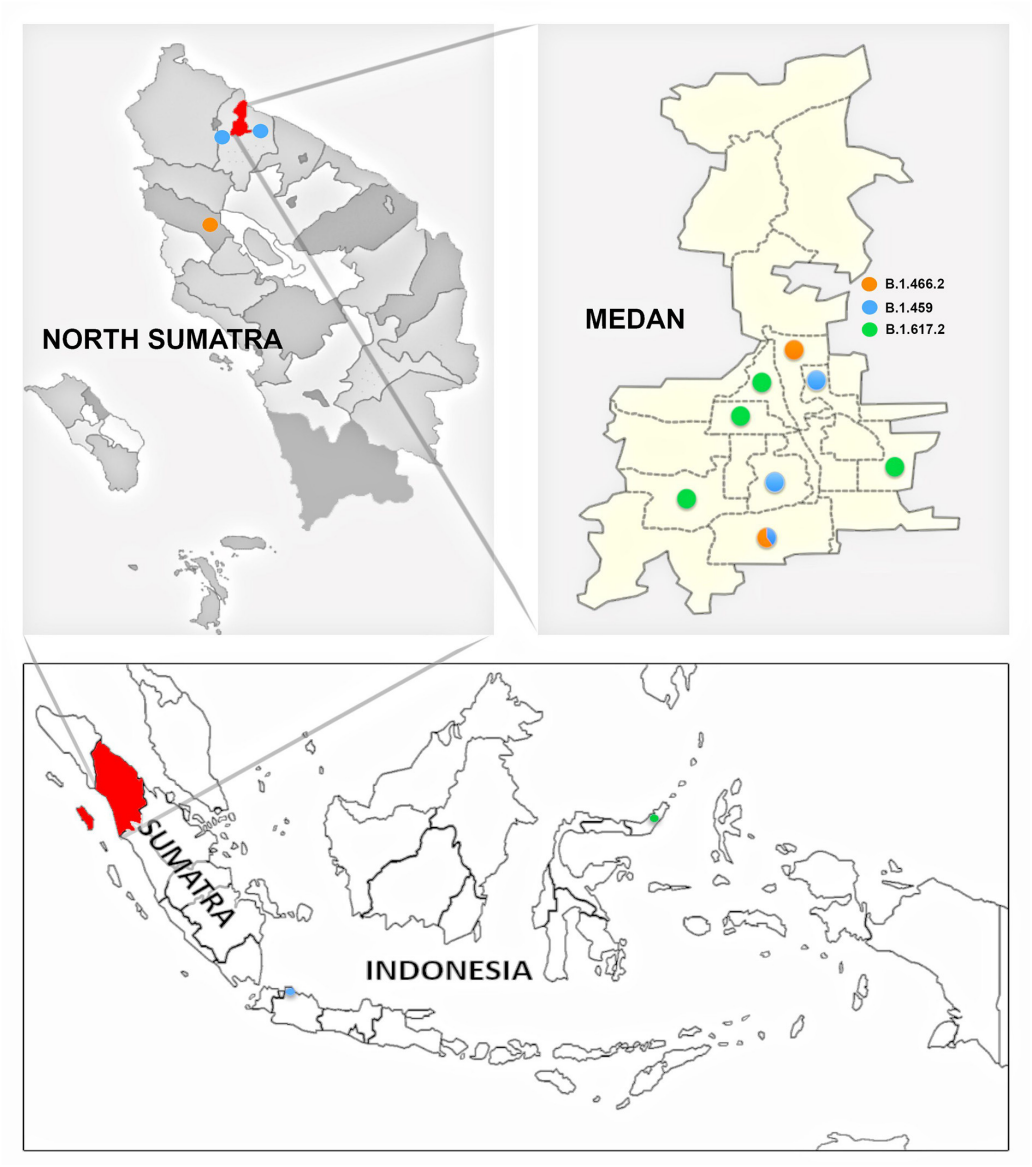
Distribution of SARS-CoV-2 variants by area of origin within or outside medan municipality. Figure legend: B.1.459 (blue); B.1.466.2 (orange); B.1.617.2 (green).

The geospatial distribution of the variants by ward in Medan Municipality based on the patient's residence (Figure 4) was also assessed. There was a tendency toward clustering of variant cases in the same ward, as only one of the wards with more than one case yielded more than one variant.

**Figure 5:**
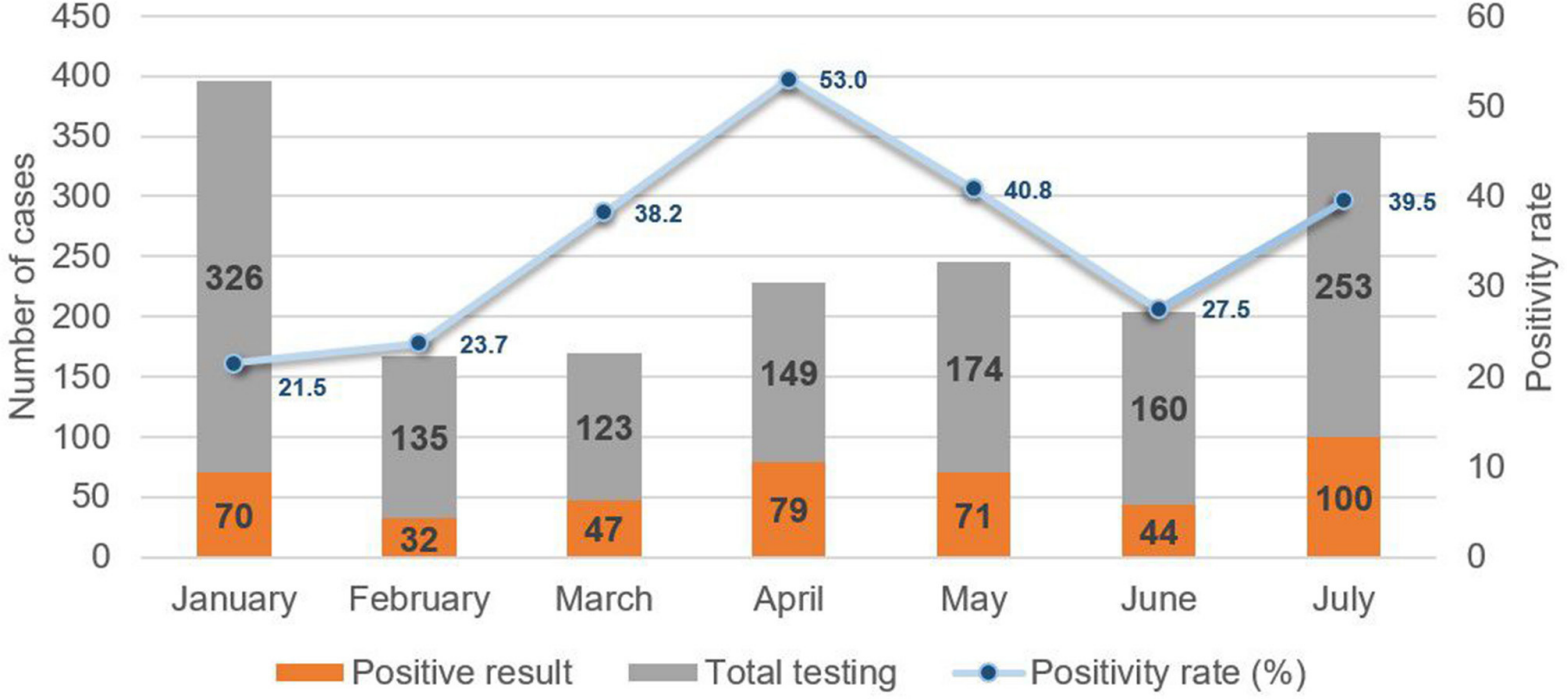
New pediatric COVID-19 cases and positivity rate from January to July 2021 at Murni Teguh Memorial Hospital.

To assess the epidemiological conditions accompanying the emergence of the Delta variant, a review of case records at MTMH from June to July 2021 revealed a rapid increase of more than twofold in the number of new pediatric COVID-19 cases. This correlated with an increase in the positivity rate from 27.5 to 39.7%, which had been preceded by a decline from 52.7% in April to 27.5% in June (Figure 5). This pattern echoed that for overall cases.

**Figure 6:**
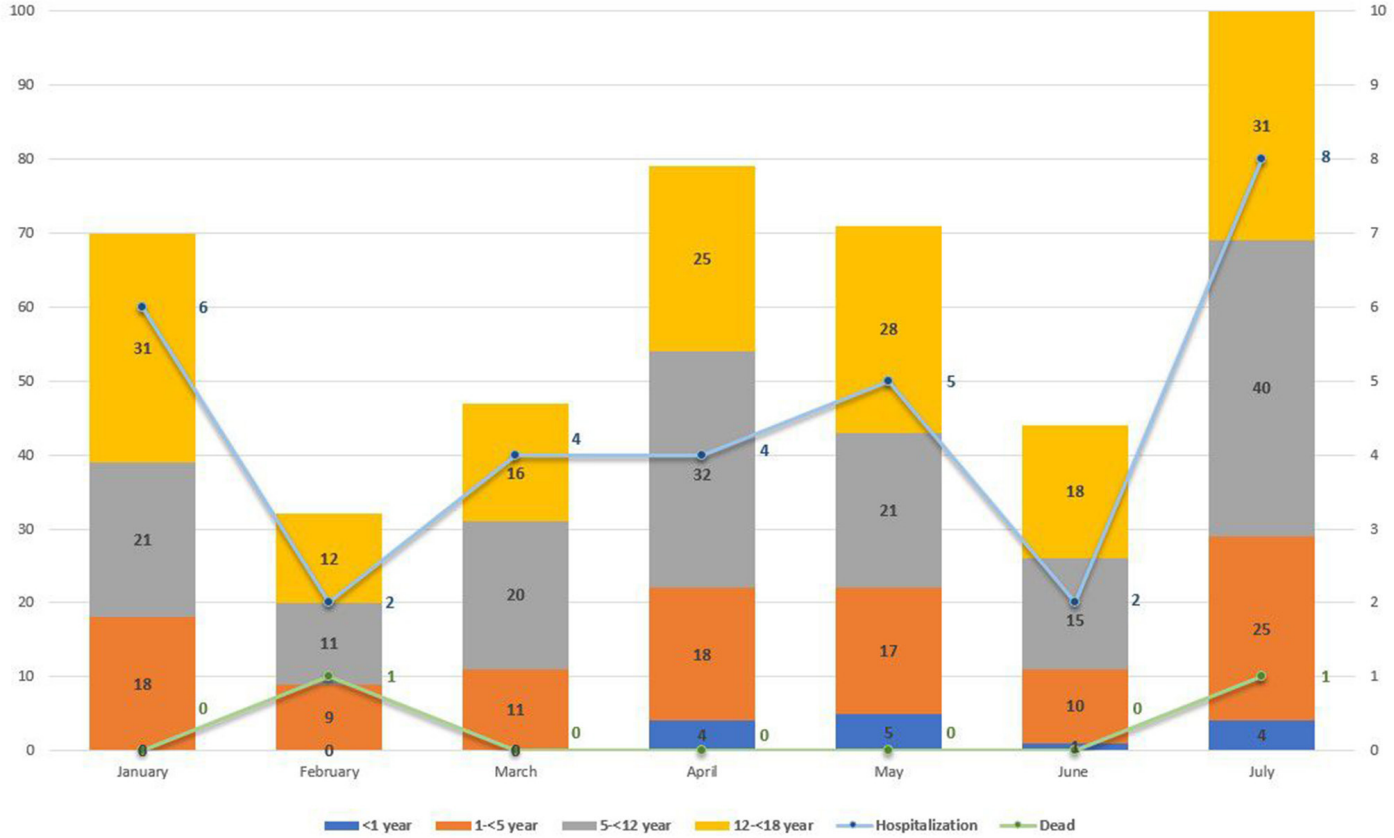
New COVID-19 paediatric cases by age group (in year) from January to July 2021 at Murni Teguh Memorial Hospital, Medan. Line graph indicates number for pediatric COVID-19 cases with hospitalization and death from January to July 2021 at Murni Teguh Memorial Hospital, Medan.

Further analysis by age group of the total new pediatric cases at MTMH from January to July 2021 showed a statistically significant change in age distribution after June 25 compared with before (χ^2^ test, *p* = 0.036) (Figure 6). Only severely or critically ill children (*n* = 31) were admitted to isolation ward at MTMH. Figure 6 reveals that the highest hospitalization rate of children infected with COVID-19 was in July (*n* = 8), as compared with other months, with two deaths during the month. Based on results from the WGS, the majority of these cases would be Delta.

Table 3 also highlights a fourfold increase in total COVID-19 pediatric cases from June to July, with the highest pediatric hospitalization rate in July. We note that from January to July 2021, the highest number of admitted cases was those 1–5 years old (*n* = 14), whereas after June 25, 2021, the highest number of pediatric COVID-19 hospitalizations was those 5–12 years old. There was one confirmed case in a child younger than 1 year who was hospitalized and died.

**TABLE 3:** Hospitalization and death by age group of children infected with SARS-CoV-2 between January and July 2021 at Murni Teguh Memorial Hospital.

	**Hospitalization**				**Total**	**Death**				**Total**
	**<1 year**	**1– <5 years**	**5– <12 years**	**12– <18 years**	**Hospitalization**	**<1 year**	**1– <5 years**	**5– <12 years**	**12– <18 years**	**Death**
January	0	3	1	2	**6**	0	0	0	0	**0**
February	0	1	0	1	**2**	0	1	0	0	**1**
March	0	2	1	1	**4**	0	0	0	0	**0**
April	0	3	0	1	**4**	0	0	0	0	**0**
May	0	1	1	3	**5**	0	0	0	0	**0**
June	0	1	1	0	**2**	0	0	0	0	**0**
July	1	3	4	0	**8**	1	0	0	0	**1**
Total	**1**	**14**	**8**	**8**	**31**	**1**	**1**	**0**	**0**	**2**

*The bold value used to highlight total number of hospitalization cases per month, total death cases per month, and total cases by age group from January to July 2021*.

**Figure 7:**
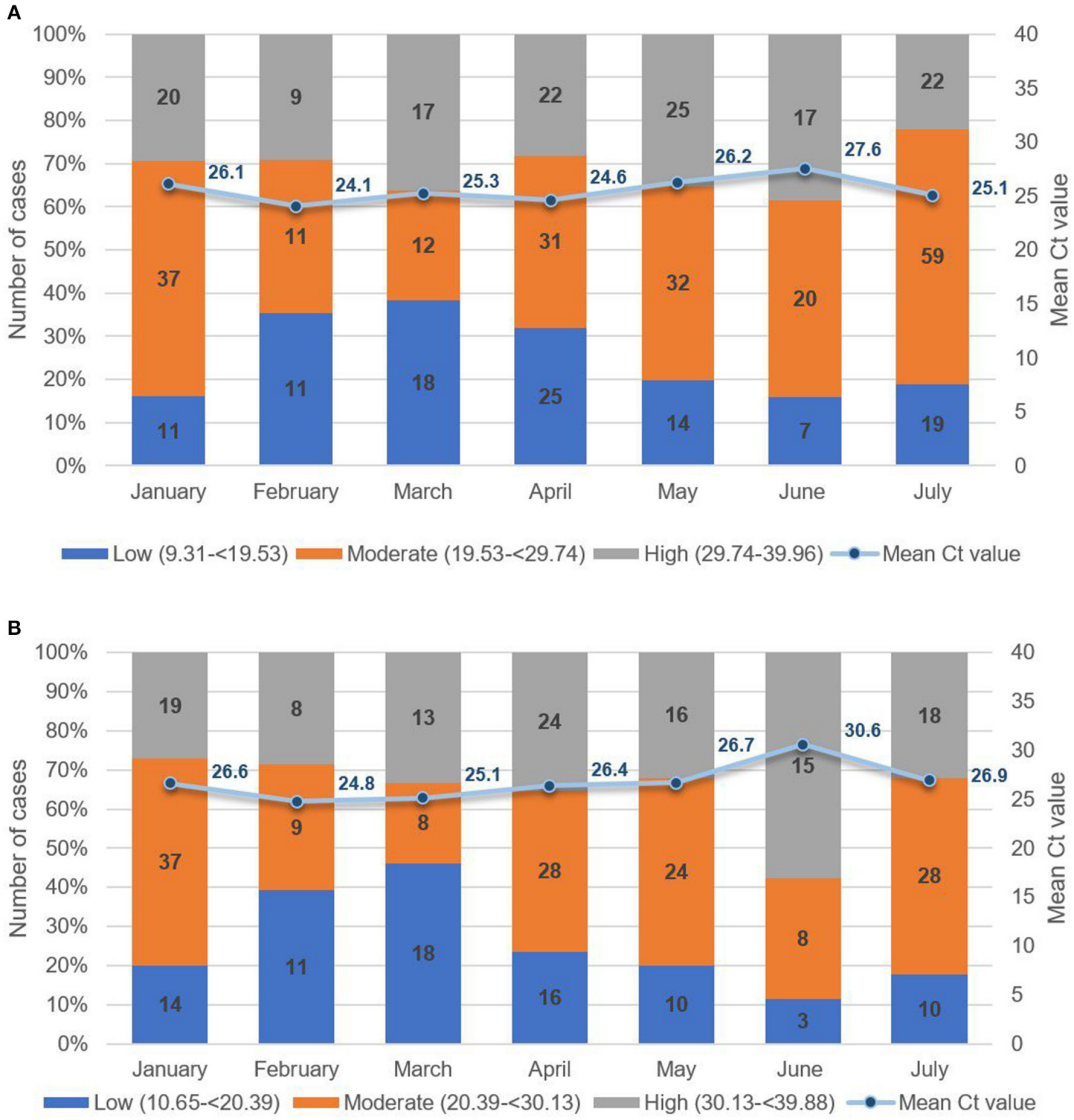
**(A)** Mean Ct values of paediatric patients for ORF or RdRP target genes low (10.65 to <20.39); moderate (20.39 to <30.13); high (30.13 to 39.88). **(B)** Mean Ct values of paediatric patients for the *N* target gene low (9.31 to <19.53); moderate (19.53 to <29.74); high (29.74 to 39.96).

To assess patterns in viral load, we analyzed Ct values from all pediatric cases from January 2021 to July 2021 (Figures 7A, B). From 1,320 suspect pediatric cases presenting at the hospital, 443 were positive for SARS-CoV-2 by RT-PCR of naso-oropharyngeal swabs. The highest number of new pediatric cases was 100 in July 2021. As seen in Figure 7A, for *ORF* and *RdRP* genes, the lowest Ct value was 10.65, and the highest was 39.88, with mean Ct values from 26.61 to 30.61. Similarly, as seen in Figure 7B, for the *N* gene, the lowest Ct value was 9.31, and the highest was 39.96, with the mean ranging from 24.05 to 27.54. Concurrent with dominance of the Delta variant in July 2021, logistic regression revealed a nonsignificant trend toward increased viral load, that is, Ct values below the median (odds ratio = 1.41, 95% CI = 0.90–2.20, *p* = 0.13), compared with previous months. Based on follow-up case tracing, four of the six pediatric cases with a Delta variant had a household contact who was RT-PCR–positive for SARS-CoV-2 on the same date of testing. Moreover, two of the six were siblings who had an adult and a child household contact. As seen in Table 4, we note a tendency wherein all 4 pediatric Delta patients with an infected adult household contact had Ct values higher, that is, lower viral load, than the adult based on pairwise dichotomous comparison for presence of a greater Ct value in the child of each child–adult dyad (Fisher exact test, *p* = 0.065).

**TABLE 4:** Results from RT-PCR for SARS-CoV-2 from six Delta pediatric cases and their household contacts at Murni Teguh Memorial Hospital Medan.

**Patient's ID**	**Age (years)**	**Date of testing**	**Ct value**	**Household contact's ID**	**Age (years)**	**Date of testing**	**Ct value**
11	14	25 June 2021	22.2	··	··	··	··
12	6	25 June 2021	24.34	Contact A	4	25 June 2021	30.2
				Contact B	35	25 June 2021	22.35
13	1	25 June 2021	25.62	Contact A	4	25 June 2021	30.2
				Contact B	35	25 June 2021	22.35
15	8	4 July 2021	17.61	··	··	··	··
17	5	5 July 21	21.63	Contact C	49	5 July 2021	20.16
18	10	11 July 2021	24.41	Contact D	34	11 July 2021	18.94

## Discussion

As of July 31, 2021, Indonesia had posted 3,917 whole-genome sequences of SARS-CoV-2 to GISAID. During the last week of July 2021, the Delta variant of concern was the dominant strain globally and in Indonesia, where it accounted for 32.4% of 510 sequences (14). To the best of our knowledge, this is the first detailed report of the Delta variant among pediatric cases in Indonesia or from an LMIC.

There were three variants detected among the 18 pediatric outpatients: two were the previously dominant lineages in Indonesia (B.1.459 and B.1.466.2), and the third was Delta (B.1.617.2). In this study, B.1.459 was present from mid-May and dominant until early June before B.1.466.2 ascended, followed by Delta becoming dominant by the end of June until mid-July when the study ended (Figure 3). The temporal change in pediatric variants was similar to the variant patterns in national data from Indonesia (2).

Identification of the six Delta variant pediatric COVID-19 cases was concurrent with the increase of new pediatric COVID-19 cases. Our results support a finding from Colorado, USA, wherein the Delta variant was highly transmissible (15) and a report from France (16) where the Delta variant presented with higher viral loads compared with other variants, perhaps partially explaining its rapid domination in Indonesia. The phylogenetic analysis showed the majority of Delta variants were closely related and, combined with the temporal tracking (Figure 3), supports higher transmission of the Delta variant in general. The pediatric genomic data of circulating SARS-CoV-2 strains reinforce infection control measures and management of children with COVID-19 (17).

The extent to which SARS-CoV-2 can be transmitted from children to other household members is unclear, including whether older persons with higher risk for severe disease are especially vulnerable to COVID-19 from children (18). Clinical data from six COVID-19 Delta pediatric cases were not recorded as they were all outpatients during specimen collection. A study in France reported that children in a family cluster of six siblings infected with the Delta variant presented with fever, asthenia, pneumonia, diarrhea, and runny nose (19). Chest computed tomography scan of pediatric patients infected with the Delta variant were milder (20), which is consistent with our observed trend of lower viral loads in pediatric cases in family clusters. However, a study in Scotland reported that infection with the Delta variant in young persons increased the risk of hospital admission by twofold compared with the Alpha variant (21). In this study, none of the children with the Delta variant were hospitalized; however, four of six children had an infected household adult contact who was tested at the same hospital and the same date. This supports the recommendation that children exposed to SARS-CoV-2 or diagnosed with COVID-19 should maintain physical distancing from other household members (22).

In this study, the Delta variant among pediatric cases was first observed in late June 2021. This coincided with the escalation in new COVID-19 cases and an increased positivity rate in North Sumatra. In addition, Indonesia had a rapid and large surge in cases, which were predominantly the Delta variant, with the highest daily new COVID-19 case number recorded on July 14, 2021 (*n* = 54,517) (23).

The Delta variant was found to infect one-third of children between 1 and 14 years of age, with five of six patients younger than 12 years. With the rapid spread of the Delta variant of COVID-19 in children, the acceleration of COVID-19 vaccination for young children is very important to protect them from the severe impact of the disease. Given that the Indonesian government currently recommends vaccination only for children 6 years or older, the findings herein support a paradigm shift in which younger children should become eligible for vaccination. All school-aged children should be considered high priority to receive vaccination to enable resumption of face-to-face teaching and learning activities. Furthermore, to sustain child education from nursery to high school, vaccination of all teachers should be mandated prior to teaching in person to avoid any transmission to their students.

Two limitations should be acknowledged in this work. First, only a small number of pediatric cases were included and sent for WGS analysis. Second, the clinical data of patients tended to be incomplete as they were from outpatient care settings.

Despite these limitations, this is the first report of the emergence of the Delta variant of concern and its dominance in pediatric cases in Indonesia. Furthermore, it supports the need for more attention to pediatric cases and spurs more action to protect children from COVID-19.

## Data Availability Statement

The datasets presented in this study can be found in online repositories. The names of the repository/repositories and accession number(s) can be found below: https://www.gisaid.org, ID EPI_ISL_3208058, EPI_ISL_3208059, EPI_ISL_3208060, EPI_ISL_3208061, EPI_ISL_3208062, EPI_ISL_3208063, EPI_ISL_3208064, EPI_ISL_3208065, EPI_ISL_3208066, EPI_ISL_3208067, EPI_ISL_3208068, EPI_ISL_3208069, EPI_ISL_3208070, EPI_ISL_3208071, EPI_ISL_3208072, EPI_ISL_3208073, EPI_ISL_3208074, EPI_ISL_3208086.

## Ethics Statement

The studies involving human participants were reviewed and approved by the Ethics Committee of the Faculty of Medicine, University of Indonesia—Cipto Mangunkusumo Hospital. Written informed consent to participate in this study was provided by the participants' legal guardian/next of kin.

## Author Contributions

RK: conceptualization, methodology, validation, formal analysis, investigation, resources, data curation, supervision, review and editing, and funding. IL: conceptualization, formal analysis, investigation, data curation, visualization, and review and editing. MK: conceptualization, methodology, software, validation, investigation, resources, data curation, and review and editing. AP: methodology, software, formal analysis, investigation, resources, data curation, visualization, review and editing, and funding. KF: conceptualization, software, formal analysis, visualization, supervision, and review and editing. MM: conceptualization, investigation, data curation, resources, and review and editing. AS: conceptualization, methodology, validation, software, formal analysis, resources, data curation, supervision, review and editing, and funding. TT: conceptualization, methodology, software, validation, formal analysis, investigation, resources, data curation, visualization, original and draft preparation, and review and editing. All authors contributed to the article and approved the submitted version.

## Funding

The sequencing was supported by Wellcome Trust Grant 222574/Z/21/Z supplement for SARS-CoV-2 genomic surveillance. The Wellcome Trust had no other role in this study.

## Conflict of Interest

The authors declare that the research was conducted in the absence of any commercial or financial relationships that could be construed as a potential conflict of interest.

## Publisher's Note

All claims expressed in this article are solely those of the authors and do not necessarily represent those of their affiliated organizations, or those of the publisher, the editors and the reviewers. Any product that may be evaluated in this article, or claim that may be made by its manufacturer, is not guaranteed or endorsed by the publisher.
